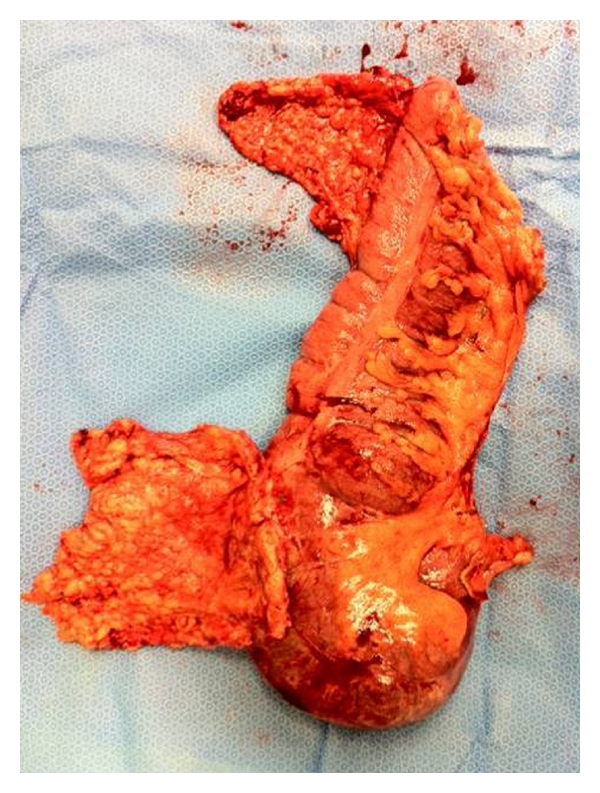# Postvaginal Delivery Caecal Volvulus and Perforation

**DOI:** 10.1155/2012/971213

**Published:** 2012-02-09

**Authors:** M. M. Abdullah Agha

**Affiliations:** Department of Obstetrics and Gynecology, Wexham Park Hospital, Slough SL2 4HL, UK

## Abstract

Intestinal obstruction is an uncommon complication of pregnancy and pueperium. It has different etiologies and voluvlus is one of the common causes. High index of suspicion is needed to diagnose it as initial presentation is nonspecific and that is critical to avoid adverse outcomes. We presented here one of these cases that followed vaginal delivery and ended with caecal perforation and hemicolectomy.

## 1. Case Report

Ms VJ, a 43-year-old Caucasian lady, in her second pregnancy, with the first baby born by emergency caesarean section in 2008. Her BMI is 25, booked at 10 weeks of gestation, with no remarkable past medical history and normal booking tests and routine scans. She had a midwifery-led care up to 31 weeks of gestation when referred to the consultant-led antenatal clinic for detecting polyhydramnios on ultrasound scan, no underlying cause was found. At 33 weeks, then she had preterm prelabour rupture of membrane (PPROM) and a conservative plan of management was followed. Three days later, she started early labour, with no evidence of infection. She was admitted then to labour ward, epidural was instituted, and then she shortly advanced to second stage. At that point, the fetal trace started showing late deceleration. Therefore, assisted delivery with Neville-Barnes forceps was done and a healthy female baby was delivered with a weight of 2225 g and normal blood gases results, she was admitted to special care unit. The patient then had a second degree perineal tear which was sutured, with estimated blood loss of 800 mL. 

Few hours later, the patient started complaining of crampy abdominal pain, with tachycardia, low blood pressure, and good urine output. Later, her pain became worse, with low grade fever, abdominal distention, tenderness, and poorer urine output. Urgent US scan then CT were done next day (as the first impression was intraperitoneal haemorrhage). The US scan showed free fluid in right hepatorenal pouch and no fluid in pouch of Douglas, while the CT scan showed 7-8 cm intrauterine haematoma and a 6 cm fusiform right levator ani muscle haematoma, extending inferiorly to the posterior right vaginal wall. In the next coming two days, the patient started showing more bowel-related signs of tympanic distention, tenderness, sluggish bowel sounds, and passing flatus only but with no nausea or vomiting. The vital observations remained the same with no further deterioration. Over the same period, Hb dropped from 114 g/L before delivery to 65 g/L, WBC increased to 20.5 ∗ 10^9^/L, Neutrophils up to 18.4 ∗ 10^9^/L, CRP soared up to 239 mg/L. The other biochemical tests of liver, renal functions, and electrolytes were generally within normal limits. Then on the second postdelivery day an abdominal X-ray was then requested and showed a very distended caecum. The general surgeons were then asked for their review and advised for conservative management plan and inserting NG tube, however, the patient's clinical condition was worsening. A second CT on the third postnatal day showed more distended caecum of 9.4 cm diameter with pneumoperitoneum, indicating perforation. Then an emergency laparotomy was planned, at theatre, a very dilated and necrotic caecal wall with volvulus and an anterior wall perforation sealed by omentum was seen (Figure 1), right hemicolectomy and end ileostomy were done with closed transverse colon brought out to surface.

The patient had a smooth postoperative recovery, discharged after eight days. Histology showed ischemic changes only with no other obvious pathology.

## 2. Discussion

Intestinal obstruction is an uncommon but serious complication of pregnancy, with reported incidence varying widely from 1 : 1500 to 1 : 66431 [1].

The common causes of mechanical intestinal obstruction complicating pregnancy and puerperium are adhesions (58%) and intussusception (5%) [1]. Volvulus is reported to be responsible for 25% of acute intestinal obstruction during pregnancy and puerperium and only 3–5% outside pregnancy [2]. Volvulus is more likely to occur at times of rapid change in the uterine size and can present during pregnancy or more commonly in puerperium [3]. Two prerequisites for development of caecal volvulus are caecal hypermobility (28% of women) and a fixed point around which rotation can occur [4].

Most frequently, perforation occurs on the anterior surface of the caecum 5 cm distal to the ileocaecal junction [5].

Initial features are nonspecific; crampy abdominal pain, tenderness and distention, nausea and vomiting, constipation, cystic mass in the mid- or upper abdomen and high-pitched bowel sounds. Signs of peritonitis may be present if perforation occurs. There may be a history of previous episodes of abdominal distention, nausea, and vomiting relieved by passage of flatus and faeces.

The differential diagnosis includes paralytic ileus, acute colonic pseudoobstruction (Ogilvie's syndrome), and intra-abdominal obstructive pathology.

Leucocytosis and elevated temperature are not consistent findings [4].

The main diagnostic aid is abdominal X-ray. Features consistent with caecal volvulus are dilated caecum in an ectopic position, single-caecal fluid level, and distended loops of small bowel often located to the right of the caecum. With no evidence of perforation or gangrene, sigmoidoscopy and a barium enema may be performed to diagnose associated distal colon obstruction and to differentiate sigmoid volvuli. Barium enema may reveal the characteristic bird-beak deformity [4].

It has been suggested that a caecal diameter of 9 cm or more indicates imminent perforation of the caecum and surgical intervention would be necessary in such cases. Moreover, failure of conservative management with continuous distention and documenting caecal perforation are other indications for surgical intervention [6].

Early operative treatment is imperative to prevent caecal perforation. Treatment should accomplish derotation, decompression, removal of devitalized segments, and anchoring to prevent recurrence. This is often accomplished by caecostomy and caecopexy. Right hemicolectomy may be necessary for large areas of perforation or gangrenous bowel [4].

Intestinal obstruction is thought to be uncommon. Therefore, recognition during antenatal and puerperium periods may be delayed. For that, high index of suspicion is essential for prompt and accurate diagnosis, as delay between presentation to diagnosis and definitive management, increases mortality rate from 8% without perforation up to 44–72% with perforation [6].

## Figures and Tables

**Figure 1:**